
JOURNAL INFORMATION
==============================
NLM Title Abbreviation: ScientificWorldJournal
Journal ID: ScientificWorldJournal
NLM Journal ID: 101131163
Journal ID: TSWJ

The Scientific World Journal

ISSN: 2356-6140
EISSN: 1537-744X
Publisher: Wiley

ARTICLE INFORMATION
==============================
PMCID: PMC6084203
PMID: 30147580
DOI: 10.1100/tsw.2001.193
Article ID: 841234
Article version: 1
Subjects: Short Report

Expression of Adenine Nucleotide Translocator Correlates with the IL-4-induced lymphocyte survival: Role of PI3K and MAPK

Jang Ji-Young
Lee Choong-Eun *
Department of Biological Science, SungKyunKwan University, Suwon, 440-746, Republic of Korea
*Choong-Eun Lee: celee@yurim.skku.ac.kr
Publication date: 2001
Electronic publication date: 2001 Dec 19
Volume: 1
First page: 100
Last page: 100
Copyright: Copyright © 2001 Ji-Young Jang and Choong-Eun Lee.
Copyright year: 2001
License: This is an open access article distributed under the Creative Commons Attribution License, which permits unrestricted use, distribution, and reproduction in any medium, provided the original work is properly cited.
License URL: https://creativecommons.org/licenses/by/3.0/

Funding: Korea Science and Engineering Foundation 1999-1-212-001-5

==============================
Miami *Nature Biotechnology* Short Reports
*TheScientificWorld* (2001) 1 (S3), 100SR
ISSN 1532-2246; DOI 10.1100/tsw.2001.193

### EXPRESSION OF ADENINE NUCLEOTIDE TRANSLOCATOR CORRELATES WITH THE IL-4-INDUCED LYMPHOCYTE SURVIVAL: ROLE OF PI3K AND MAPK

Ji-Young Jang and Choong-Eun Lee*

Department of Biological Science, SungKyunKwan University, Suwon, 440-746, Korea
* celee@yurim.skku.ac.kr

**INTRODUCTION.** Cytokines regulate the survival and apoptosis of lymphocytes during T cell proliferation and differentiation. Since Th1 and Th2 cytokines are thought to play an important role in the induction and response of Th1 vs. Th2 cells, we have been interested to identify novel factors regulating Th1 vs. Th2 response via modulation of T cell apoptosis and survival.

**MATERIALS AND METHODS.** We have employed DD-PCR techniques to screen novel genes whose expression is differentially regulated by Th1 and Th2 cytokines in human lymphocytes. One of the selected DD-PCR products was adenine nucleotide translocator(ANT3). The mRNA expression pattern by various Th1 and Th2 cytokines was analyzed by Northern blot and RT-PCR in B, T, and monocytic cells. The apoptosis study was performed using DNA fragmentation analysis and $DiOC_6$ and PI staining by flow cytometry.

**RESULTS.** ANT(3) was identified as a gene induced by IL-4 and counter-regulated by IFN-r , a prototypic Th2 and Th1 cytokine. Such counter-regulation was found primarily in T cells, but not in B cells or monocytes. We observed that under the condition in which IL-4 rescued CD4+ lymphocytes from dexamethasone-induced apoptosis, there was a concomittant increase in the ANT mRNA expression by IL-4. While IL-4 induced PI3K and MAPK activity in these cells, inhibitors of PI3K and MAPK both effectively suppressed the IL-4-induced lymphocyte survival and ANT expression. The up-regulation of ANT mRNA level by IL-4 seems to involve, at least in part, an increase in ANT mRNA stability, since the effect of IL-4 was more prominently observed upon using 3'UTR probe than the coding region probe of ANT. In addition, IGF-1, which activates PI3K and MAPK via IRS-1, not only induced the ANT gene expression, but also agumented the IL-4-induced ANT mRNA up-regulation in T cells.

**CONCLUSION.** While the function of ANT has been well recognized for maintaining cellular energy supply and metabolitic regulation, recent studies proposed a role of ANT as an apoptotic regulator by interacting with Bax. Our present findings indicate that the IL-4-induced lymphocyte rescue from apoptosis is mediated via PI3K and MAPK, down-stream effectors of IRS-1/2 through the up-regulation of ANT mRNA stability, suggesting a role of ANT in the IL-4-mediated Th cell survival.

**ACKNOWLEDGEMENT.** This work was supported by grants from KOSEF(1999-1-212-001-5), BK21 program, and 21C Frontier Human Genome Research Project.

**REFERENCES.**

1. Murphy, K.M., Ouyang, W., Farrar, D.J., et al. (2000) Annu. Rev. Immunol. 18, 451-494
2. Marzo, I., Brenner, C., Zamzami, N., et al. (1998) Science 281, 2027-2031
